# Crystal structure of ethyl 2-(4-chloro­anilino)acetate

**DOI:** 10.1107/S1600536814018297

**Published:** 2014-08-16

**Authors:** Jamila Bouali, Abderrafia Hafid, Mostafa Khouili, Mohamed Saadi, El Mostafa Ketatni

**Affiliations:** a Laboratoire de Chimie Organique et Analytique, Université Sultan Moulay Slimane, Faculté des Sciences et Techniques, BP 523, 23000 Béni-Mellal, Morocco; b Laboratoire de Chimie du Solide Appliquée, Faculté des Sciences, Université Mohammed V-Agdal, Avenue Ibn Battouta, BP 1014, Rabat, Morocco; c Laboratoire de Spectrochimie Applique et Environnement, Université Sultan Moulay Slimane, Faculté des Sciences et Techniques, BP 523, 23000 Béni-Mellal, Morocco

**Keywords:** crystal structure, ethyl 2-(4-chloro­anilino)acetate, syndone derivatives, biological activity, hydrogen bonding

## Abstract

The title compound, C_10_H_12_ClNO_2_, is close to planar (r.m.s. deviation for the 14 non-H atoms = 0.053 Å). In the crystal, inversion dimers linked by pairs of N—H⋯O_c_ (c = carbox­yl) hydrogen bonds generate *R*_2_^2^(10) loops.

## Related literature   

For the biological activity of sydnone derivatives, see: Satheesha Rai *et al.* (2008 ▶); Patel & Patel (2012 ▶). For an overview of sydnone derivatives, see: Asundaria *et al.* (2010 ▶); Ding *et al.* (2013 ▶); Fadda & Elattar (2012 ▶). For a related structure, see: Zhang *et al.* (2010 ▶).
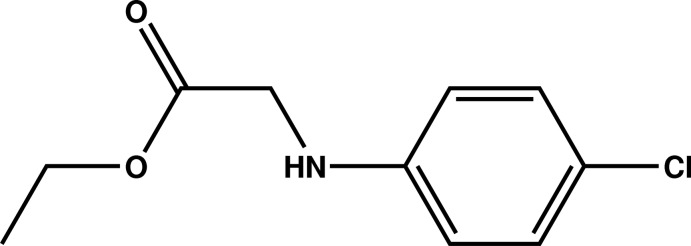


## Experimental   

### Crystal data  

C_10_H_12_ClNO_2_
*M* *_r_* = 213.66
Triclinic, 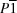

*a* = 5.373 (5) Å
*b* = 7.575 (7) Å
*c* = 14.127 (12) Å
α = 75.83 (4)°
β = 87.73 (3)°
γ = 72.99 (3)°
*V* = 532.7 (9) Å^3^
*Z* = 2
Mo *K*α radiation
μ = 0.33 mm^−1^
*T* = 296 K
0.40 × 0.36 × 0.29 mm

### Data collection  

Bruker X8 APEX CCD diffractometer
Absorption correction: multi-scan (*SADABS*; Bruker, 2009) ▶ *T*_min_ = 0.693, *T*_max_ = 0.747
3672 measured reflections
2361 independent reflections
1718 reflections with *I* > 2σ(*I*)
*R*_int_ = 0.018

### Refinement  

*R*[*F* ^2^ > 2σ(*F* ^2^)] = 0.046
*wR*(*F* ^2^) = 0.136
*S* = 1.03
2361 reflections
127 parameters
H-atom parameters constrained
Δρ_max_ = 0.23 e Å^−3^
Δρ_min_ = −0.25 e Å^−3^

### 

Data collection: *APEX2* (Bruker, 2009 ▶); cell refinement: *SAINT* (Bruker, 2009 ▶); data reduction: *SAINT*; program(s) used to solve structure: *SHELXS97* (Sheldrick, 2008 ▶); program(s) used to refine structure: *SHELXL97* (Sheldrick, 2008 ▶); molecular graphics: *ORTEP-3 for Windows* (Farrugia, 2012 ▶); software used to prepare material for publication: *PLATON* (Spek, 2009 ▶) and *publCIF* (Westrip, 2010 ▶).

## Supplementary Material

Crystal structure: contains datablock(s) I. DOI: 10.1107/S1600536814018297/hb7269sup1.cif

Structure factors: contains datablock(s) I. DOI: 10.1107/S1600536814018297/hb7269Isup2.hkl

Supporting information file. DOI: 10.1107/S1600536814018297/hb7269Isup3.cml

**Fig. 1.**
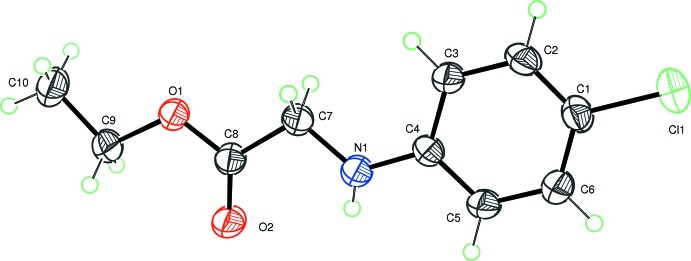
Molecular structure of the title compound with displacement ellipsoids drawn at the 50% probability level.

. DOI: 10.1107/S1600536814018297/hb7269fig1.tif
Mol­ecular structure of the title compound with displacement ellipsoids drawn at the 50% probability level.

**Fig. 2.**
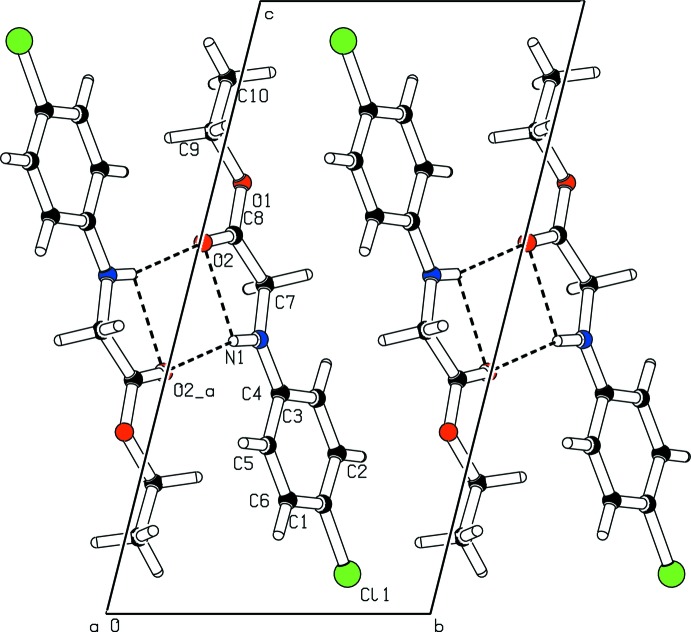
Part of the crystal structure of the title compound, showing hydrogen-bonded (dashed lines) dimers.

. DOI: 10.1107/S1600536814018297/hb7269fig2.tif
Part of the crystal structure of the title compound, showing hydrogen-bonded (dashed lines) dimers.

CCDC reference: 1018685

Additional supporting information:  crystallographic information; 3D view; checkCIF report

## Figures and Tables

**Table 1:** Hydrogen-bond geometry (Å, °)

*D*—H⋯*A*	*D*—H	H⋯*A*	*D*⋯*A*	*D*—H⋯*A*
N1—H1⋯O2^i^	0.82	2.37	3.172 (3)	165

Symmetry code: (i) 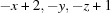.

## supplementary crystallographic information

### S1. Comment

The title compound was synthesized as a precursor for the preparation of biological sydnone derivatives (Satheesha Rai *et al.*, 2008; Patel & Patel, 2012, Asundaria *et al.*, 2010; Ding *et al.*, 2013; Fadda & Elattar, 2012). In the title molecule (Fig. 1), there are two planar subunits *viz*. the chlorophenyl amine (C1-C6N1Cl1) and ethyl acetate (C7C8O2O1C9C10) groups. The chlorophenyl amino ring is inclined at angles of 2.01 (9)° to the ethyl acetate groups. The substituted amino substituent is in an extended conformation with an N—C—C—O torsion angle of 179.8 (2)°. In the crystal structure, pairs of molecules are connected by intermolecular N—H···O hydrogen bonds to form centrosymmetric dimers (Fig. 2). Bond lengths and angles (Table 2) are compatible with those found in a related compound (Zhang *et al.*, 2010).

### S2. Experimental

A solution of corresponding 4-chloroaniline (2.22 g, 0.0174 mol), anhydrous sodium acetate (2.14 g, 0.0261 mol, 1.5 equiv), and ethyl chloroacetate (2.13 g, 0.0174 mol) was heated under reflux for 18 h. Then cold water (70 ml) was added with stirring and cooling in ice bath. The reaction mixture was neutralized with NaHCO_3_ and then extracted with dichloromethane (3× 25 ml). The dichloromethane extracts were dried over anhydrous Na_2_SO_4_ and concentrated. The crude product was purified by column chromatography on silica gel using hexane/ethyl acetate (9/1) as eluent. Yellow blocks were isolated when the solvent was allowed to evaporate

### S3. Refinement

All H atoms could be located in a difference Fourier map. However, they were placed in calculated positions with C–H = 0.93–0.97 Å; N—H = 0.86 Å, and refined as riding on their parent atoms with *U*_iso_(H) = 1.2 *U*_eq_ for aromatic, ethylene C—H, N–H and *U*_iso_(H) = 1.5 *U*_eq_ for methyl. One outlier (0 0 1) was omitted in the last cycles of refinement.

### Crystal data

**Table:**

C_10_H_12_ClNO_2_	*Z* = 2
*M**_r_* = 213.66	*F*(000) = 224
Triclinic, *P*1	*D*_x_ = 1.332 Mg m^−^^3^
Hall symbol: -p 1	Mo *K*α radiation, λ = 0.71073 Å
*a* = 5.373 (5) Å	Cell parameters from 2361 reflections
*b* = 7.575 (7) Å	θ = 2.9–27.5°
*c* = 14.127 (12) Å	µ = 0.33 mm^−^^1^
α = 75.83 (4)°	*T* = 296 K
β = 87.73 (3)°	Block, yellow
γ = 72.99 (3)°	0.40 × 0.36 × 0.29 mm
*V* = 532.7 (9) Å^3^	

### Data collection

**Table:**

Bruker X8 APEX CCD diffractometer	2361 independent reflections
Radiation source: fine-focus sealed tube	1718 reflections with *I* > 2σ(*I*)
Graphite monochromator	*R*_int_ = 0.018
φ and ω scans	θ_max_ = 27.5°, θ_min_ = 2.9°
Absorption correction: multi-scan (*SADABS*; Bruker, 2009)	*h* = −6→6
*T*_min_ = 0.693, *T*_max_ = 0.747	*k* = −9→7
3672 measured reflections	*l* = −18→10

### Refinement

**Table:**

Refinement on *F*^2^	Primary atom site location: structure-invariant direct methods
Least-squares matrix: full	Secondary atom site location: difference Fourier map
*R*[*F*^2^ > 2σ(*F*^2^)] = 0.046	Hydrogen site location: inferred from neighbouring sites
*wR*(*F*^2^) = 0.136	H-atom parameters constrained
*S* = 1.03	*w* = 1/[σ^2^(*F*_o_^2^) + (0.0633*P*)^2^ + 0.1405*P*] where *P* = (*F*_o_^2^ + 2*F*_c_^2^)/3
2361 reflections	(Δ/σ)_max_ < 0.001
127 parameters	Δρ_max_ = 0.23 e Å^−^^3^
0 restraints	Δρ_min_ = −0.25 e Å^−^^3^

### Special details

**Table:**

Geometry. All e.s.d.'s (except the e.s.d. in the dihedral angle between two l.s. planes) are estimated using the full covariance matrix. The cell e.s.d.'s are taken into account individually in the estimation of e.s.d.'s in distances, angles and torsion angles; correlations between e.s.d.'s in cell parameters are only used when they are defined by crystal symmetry. An approximate (isotropic) treatment of cell e.s.d.'s is used for estimating e.s.d.'s involving l.s. planes.
Refinement. Refinement of *F*^2^ against all reflections. The weighted *R*-factor *wR* and goodness of fit *S* are based on *F*^2^, conventional *R*-factors *R* are based on *F*, with *F* set to zero for negative *F*^2^. The threshold expression of *F*^2^ > σ(*F*^2^) is used only for calculating *R*-factors(gt) *etc*. and is not relevant to the choice of reflections for refinement. *R*-factors based on *F*^2^ are statistically about twice as large as those based on *F*, and *R*- factors based on all data will be even larger.

### Fractional atomic coordinates and isotropic or equivalent isotropic displacement parameters (Å^2^)

**Table:**

	*x*	*y*	*z*	*U*_iso_*/*U*_eq_	
C1	0.2530 (4)	0.5821 (3)	0.17905 (14)	0.0493 (5)	
C2	0.1089 (4)	0.5877 (3)	0.26135 (14)	0.0493 (5)	
H2	−0.0633	0.6634	0.2564	0.059*	
C3	0.2208 (3)	0.4803 (3)	0.35168 (14)	0.0460 (4)	
H3	0.1234	0.4845	0.4074	0.055*	
C4	0.4789 (3)	0.3659 (2)	0.35964 (13)	0.0408 (4)	
C5	0.6209 (3)	0.3653 (3)	0.27463 (14)	0.0466 (4)	
H5	0.7939	0.2912	0.2788	0.056*	
C6	0.5104 (4)	0.4718 (3)	0.18512 (14)	0.0511 (5)	
H6	0.6073	0.4700	0.1292	0.061*	
C7	0.4644 (3)	0.2344 (3)	0.53739 (13)	0.0434 (4)	
H7A	0.3204	0.1854	0.5300	0.052*	
H7B	0.3945	0.3563	0.5536	0.052*	
C8	0.6487 (3)	0.0983 (2)	0.61773 (13)	0.0421 (4)	
C9	0.6934 (4)	−0.0457 (3)	0.78700 (13)	0.0494 (5)	
H9A	0.8456	−0.0068	0.7962	0.059*	
H9B	0.7510	−0.1735	0.7770	0.059*	
C10	0.5267 (5)	−0.0410 (4)	0.87449 (15)	0.0683 (6)	
H10A	0.6254	−0.1259	0.9314	0.102*	
H10B	0.3772	−0.0802	0.8646	0.102*	
H10C	0.4708	0.0861	0.8836	0.102*	
N1	0.5998 (3)	0.2584 (3)	0.44760 (12)	0.0593 (5)	
H1	0.7422	0.1814	0.4453	0.071*	
O1	0.5353 (2)	0.08512 (18)	0.70336 (9)	0.0470 (3)	
O2	0.8685 (3)	0.0108 (2)	0.60581 (10)	0.0620 (4)	
Cl1	0.11032 (12)	0.71314 (11)	0.06510 (4)	0.0837 (3)	

### Atomic displacement parameters (Å^2^)

**Table:**

	*U*^11^	*U*^22^	*U*^33^	*U*^12^	*U*^13^	*U*^23^
C1	0.0484 (10)	0.0478 (10)	0.0434 (10)	−0.0104 (8)	−0.0067 (8)	0.0011 (8)
C2	0.0367 (9)	0.0459 (10)	0.0546 (11)	−0.0012 (8)	−0.0042 (8)	−0.0048 (8)
C3	0.0408 (9)	0.0471 (10)	0.0415 (10)	−0.0031 (8)	0.0042 (7)	−0.0071 (8)
C4	0.0377 (9)	0.0375 (9)	0.0417 (9)	−0.0052 (7)	−0.0013 (7)	−0.0062 (7)
C5	0.0359 (9)	0.0472 (10)	0.0480 (10)	−0.0025 (8)	0.0044 (7)	−0.0079 (8)
C6	0.0487 (10)	0.0553 (11)	0.0429 (10)	−0.0120 (9)	0.0060 (8)	−0.0048 (8)
C7	0.0422 (9)	0.0403 (9)	0.0413 (9)	−0.0030 (7)	0.0006 (7)	−0.0093 (7)
C8	0.0443 (10)	0.0376 (9)	0.0398 (9)	−0.0044 (8)	0.0018 (7)	−0.0101 (7)
C9	0.0471 (10)	0.0517 (11)	0.0402 (10)	−0.0044 (8)	−0.0025 (8)	−0.0058 (8)
C10	0.0728 (15)	0.0781 (15)	0.0406 (11)	−0.0065 (12)	0.0067 (10)	−0.0096 (10)
N1	0.0437 (9)	0.0669 (11)	0.0412 (9)	0.0132 (8)	0.0029 (7)	−0.0013 (8)
O1	0.0440 (7)	0.0480 (7)	0.0375 (7)	0.0006 (5)	0.0023 (5)	−0.0068 (5)
O2	0.0479 (8)	0.0668 (9)	0.0468 (8)	0.0120 (7)	0.0062 (6)	−0.0042 (7)
Cl1	0.0720 (4)	0.1006 (5)	0.0500 (4)	−0.0037 (3)	−0.0123 (3)	0.0101 (3)

### Geometric parameters (Å, º)

**Table:**

C1—C2	1.373 (3)	C7—C8	1.500 (3)
C1—C6	1.384 (3)	C7—H7A	0.9700
C1—Cl1	1.742 (2)	C7—H7B	0.9700
C2—C3	1.385 (3)	C8—O2	1.202 (2)
C2—H2	0.9300	C8—O1	1.328 (2)
C3—C4	1.396 (3)	C9—O1	1.453 (2)
C3—H3	0.9300	C9—C10	1.499 (3)
C4—N1	1.374 (2)	C9—H9A	0.9700
C4—C5	1.397 (3)	C9—H9B	0.9700
C5—C6	1.372 (3)	C10—H10A	0.9600
C5—H5	0.9300	C10—H10B	0.9600
C6—H6	0.9300	C10—H10C	0.9600
C7—N1	1.436 (3)	N1—H1	0.8201
			
C2—C1—C6	120.88 (18)	C8—C7—H7B	109.8
C2—C1—Cl1	119.85 (16)	H7A—C7—H7B	108.2
C6—C1—Cl1	119.27 (16)	O2—C8—O1	124.56 (17)
C1—C2—C3	119.83 (18)	O2—C8—C7	124.41 (17)
C1—C2—H2	120.1	O1—C8—C7	111.03 (16)
C3—C2—H2	120.1	O1—C9—C10	107.18 (17)
C2—C3—C4	120.43 (17)	O1—C9—H9A	110.3
C2—C3—H3	119.8	C10—C9—H9A	110.3
C4—C3—H3	119.8	O1—C9—H9B	110.3
N1—C4—C3	122.69 (17)	C10—C9—H9B	110.3
N1—C4—C5	119.02 (17)	H9A—C9—H9B	108.5
C3—C4—C5	118.27 (16)	C9—C10—H10A	109.5
C6—C5—C4	121.34 (17)	C9—C10—H10B	109.5
C6—C5—H5	119.3	H10A—C10—H10B	109.5
C4—C5—H5	119.3	C9—C10—H10C	109.5
C5—C6—C1	119.24 (18)	H10A—C10—H10C	109.5
C5—C6—H6	120.4	H10B—C10—H10C	109.5
C1—C6—H6	120.4	C4—N1—C7	123.16 (16)
N1—C7—C8	109.52 (16)	C4—N1—H1	116.6
N1—C7—H7A	109.8	C7—N1—H1	117.7
C8—C7—H7A	109.8	C8—O1—C9	116.23 (15)
N1—C7—H7B	109.8		

### Hydrogen-bond geometry (Å, º)

**Table:**

*D*—H···*A*	*D*—H	H···*A*	*D*···*A*	*D*—H···*A*
N1—H1···O2^i^	0.82	2.37	3.172 (3)	165

Symmetry code: (i) −*x*+2, −*y*, −*z*+1.
