# Supplementary figures and images for: Crystal structure of ethyl 2-(4-chloro­anilino)acetate
Source: Acta Crystallogr Sect E Struct Rep Online. 2014 Aug 16;70(Pt 9):o1017. doi: 10.1107/S1600536814018297 (PMC4186106; doi:10.1107/S1600536814018297)

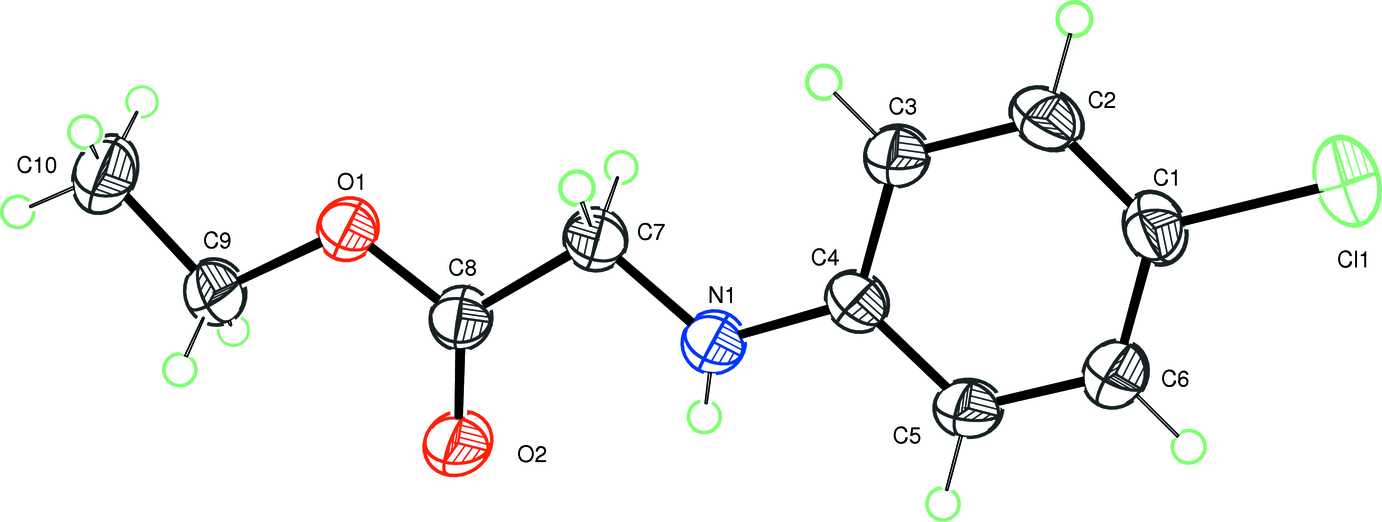

Supplement: Supplementary file 4 [file e-70-o1017-fig1.tif]

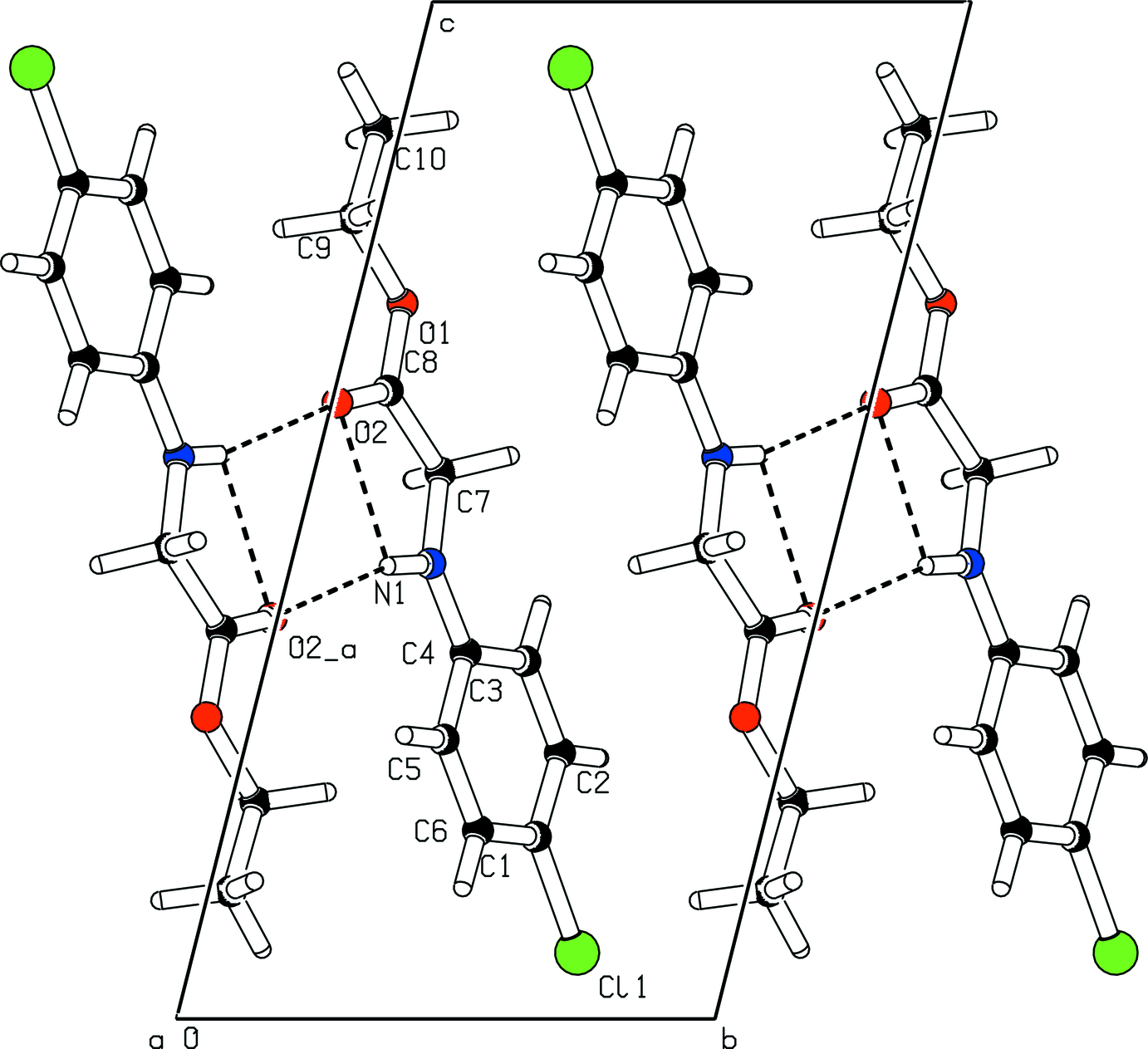

Supplement: Supplementary file 5 [file e-70-o1017-fig2.tif]
